# Predictive role of corneal Q-value differences between nasal–temporal and superior–inferior quadrants in orthokeratology lens decentration

**DOI:** 10.1097/MD.0000000000005837

**Published:** 2017-01-13

**Authors:** Juan Li, Cheng Yang, Wenjuan Xie, Guanrong Zhang, Xue Li, Shujun Wang, Xiaohong Yang, Jin Zeng

**Affiliations:** a Department of Ophthalmology, Guangdong Eye Institute, Guangdong General Hospital, Guangdong Academy of Medical Sciences, Guangzhou, Guangdong, China; b Health Management Center, Guangdong General Hospital, Guangdong Academy of Medical Sciences, Guangzhou, Guangdong, China.

**Keywords:** corneal Q-value, eccentricity, horizontal keratorefractive power, lens decentration, lens-decentration, myopia, orthokeratology

## Abstract

**Background:**

To investigate the association between pretreatment corneal parameters and orthokeratology lens decentration.

**Methods:**

This retrospective study included a total of 108 eyes in 60 myopia patients, who were divided into a lens-decentration and a control group. Various pretreatment corneal parameters were analyzed by receiver operating characteristic curves (ROC curves), including corneal horizontal and vertical curvatures, diopter, corneal eccentricity (E-value), asphericity (Q-value), diameter, and astigmatism, to establish a reliable predictive model for orthokeratology lens decentration.

**Results:**

The temporal and inferior quadrants are preferential sides for lens decentration, which was associated with the occurrence of complications such as ghosting and corneal epithelial staining. By further analysis, we revealed lower corneal horizontal curvature and much higher corneal Q-value differences between the nasal–temporal and superior–inferior quadrants in the lens-decentration group compared to the control group (*P* < 0.05). ROC curve analysis showed that the sum of Q-value differences between the nasal–temporal and superior–inferior quadrants was more sensitive than any other corneal parameters in predicting lens decentration, with an area under the curve of 0.778 and a truncation point of 0.3 (*P* < 0.001).

**Conclusion:**

The sum of pretreatment corneal Q-value differences between nasal–temporal and superior–inferior quadrants is a convenient and reliable predictor for orthokeratology lens decentration.

## 1 Introduction

Orthokeratology is a refractive correction technology that uses rigid contact lenses to temporarily reduce the refractive error of myopia by flattening the cornea and reducing the corneal curvature during sleep.^[1,2]^ Proposed mechanisms are hydraulic forces within the tear lens^[3]^ or pressure of the eye lids and lens during sleep.^[4]^

The biggest advantage of orthokeratology is that it rapidly improves visual acuity without the need for surgery or glasses.^[5]^ Since the 1990s, the introduction of contact lens material with high oxygen permeability, computerized corneal topography, and computer-controlled precision lathes have brought to orthokeratology lens technology a revolutionary breakthrough.^[6]^ To date, orthokeratology has been extensively used by ophthalmologists and optometrists around the world and shown to be an effective and safe method for the treatment of myopia.^[7]^

Despite its many advantages, orthokeratology also has some severe complications, of which lens decentration is one of the most common.^[2]^ Since the lens closely binds to the cornea overnight, lens decentration will not only reduce the therapeutic efficacy, but also result in acute or chronic complications in both the structure and function of the cornea.^[8–10]^

The major complications associated with lens decentration include ghosting, double vision, corneal epithelial staining, corneal central islands, and indentation. However, so far, the exact cause of lens decentration and an effective preventive measure for it remain unclear.^[2]^

Although the individual difference in corneal shape is considerably significant, the design and manufacture of orthokeratology lens is currently far from individualized. Thus, it is possible that complex corneal morphology and the lack of individualized lens underlie the occurrence of lens decentration.^[2,11]^ In the present study, we compared a series of pretreatment corneal parameters between eyes, with and without orthokeratology lens decentration. We revealed that eyes with lens decentration had much higher corneal asphericity (Q-value) differences between the nasal–temporal and superior–inferior quadrants than eyes without lens decentration. This significant asymmetry of corneal Q-value explains why orthokeratology lenses easily shift to the temporal and inferior quadrants. Moreover, our data also demonstrated that the sum of pretreatment corneal Q-value differences between nasal–temporal and superior–inferior quadrants was a convenient and reliable predictor for orthokeratology lens decentration in the clinic.

## 2 Patients and methods

### 2.1 Study subjects

This retrospective study included a total of 108 eyes in 60 Han Chinese patients with myopia who underwent orthokeratology from January 2013 to June 2014 in Guangdong General Hospital.

Half of them were diagnosed with lens decentration and included in the lens-decentration group, while the other half were normal after orthokeratology and included in the control group (Table 1). Age, place of residence, and gender were comparable between the 2 groups of patients. Structural lesions were excluded for each patient via ophthalmic slit lamp microscopy and ophthalmofundoscopy. Written informed consent was obtained from all patients or their guardians before their participation in our study. This study was approved by the institutional research ethics committee of Guangdong General Hospital, in compliance with the Declaration of Helsinki.

**Table 1:**
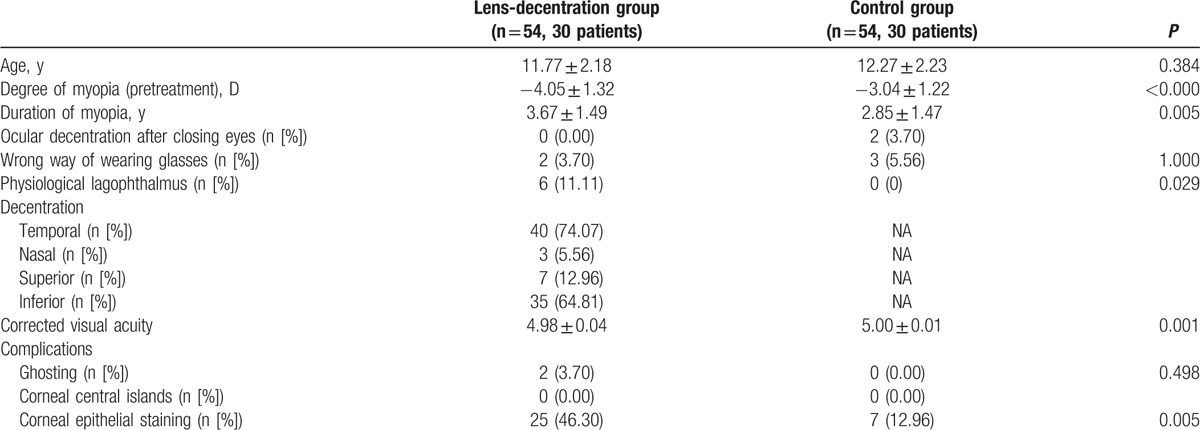
Comparison of the baseline characteristics and results of orthokeratology for patients from both groups.

### 2.2 Pretreatment eye examinations and correction

A series of examinations were performed in patients from both groups before orthokeratology, including unaided and best corrected visual acuity, subjective refraction and mydriatic retinoscopy, intraocular pressure as well as determinations of corneal topography and curvature and diameters via Pentagram topographer (Oculus; Wetzlar, Germany) measurements. The Pentacam tomographer produces a 3-dimensional elevation model of the anterior eye segment. This model is the basis for all additional calculations. The corneal Q-value was automatically calculated by the Pentacam software according to the formula Q = −E^2^ (E-value = eccentricity). A Q-value less than 0 is considered prolate, and a Q-value greater than 0 is oblate. All Q-values included nasal, temporal, inferior, and superior quadrant data.

The same experienced operator performed all Pentacam eye measurements on each patient.

The results of these examinations were collected and further analyzed.

Depending on the patient's choice, based on the diopters after mydriatic retinoscopy, corneal curvature, corneal E-value, and corneal topography, the lens power was determined and optimal orthokeratology lenses were prescribed after several try-ons. During the next 6 months, all patients were taught to wear rigid gas permeable contact lens (Autek China Inc., Hefei, Anhui, China) for overnight orthokeratology every night for 8 to 10 hours. The contact lenses, of which the optical center thickness was 0.24 mm, were made from Boston XO (Boston, Massachusetts, USA) (DK: 100 × 10^−11^ cm^2^ mLO_2_/[s mL mm Hg]) and designed in a specific geometrical shape as shown in Table 2.

**Table 2:**
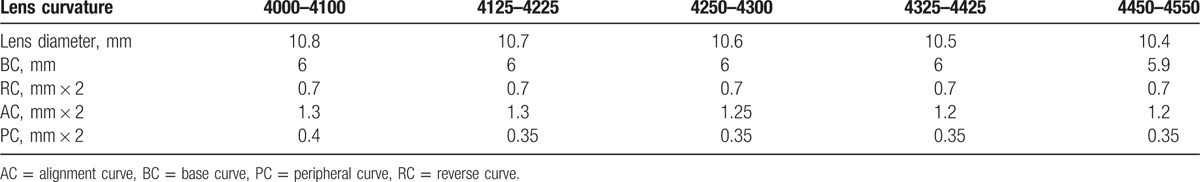
Orthokeratology lens designs.

### 2.3 Posttreatment examinations

Follow-up examinations for each patient were completed at 1 day, 1 week, 2 weeks, 3 weeks, 1 month, 3 months, 6 months, and every 3 months after 6 months. Examinations included unaided and best corrected visual acuity, subjective refraction, corneal topography, and lens decentration. Measurement of lens decentration was established as follows: according to the compartmentation of the corneal topography, optical zones ranged from the corneal apex to where the keratometry values changed within 1 D. The optical zone was limited to less than 2 types of colors in the absolute scale. The center of the optical zone after orthokeratology fitting was determined by placing a piece of transparent paper on the corneal topography and marking the farthest 4 edges of the optical zone in the X- and Y-axes with a fine-tipped pen. The pupillary center as determined by corneal topography was used as the reference point using pupil-finding software. The center of the optical zone was estimated to be the intersecting point of these 4 points. The distance between the pupillary center and the center of the optical zone was measured, and then the direction from the reference point was calculated in degree scales. The amount of decentration of orthokeratology lens was measured by finding the distance between the center of the optic zone and the pupil center.^[12]^ Considering that lens decentration tends to stabilize within 6 months after orthokeratology, only patients with a decentration larger than 1 mm after 6 months were included in the lens-decentration group and others were included in the control group. Measurements of unaided visual acuity were accomplished within 6 months of orthokeratology.

### 2.4 Statistics

Statistical analysis was performed using IBM SPSS Statistics for Windows (version 20.0, IBM Corp.; Armonk, NY) and MEDCALC (version 12.0; Medcalc, Mariakerke, Belgium). A *t* test and/or a chi-square test was used to compare the parameters between the 2 groups, and receiver operating characteristic curvature (ROC curvature) analysis was adopted to evaluate their predictive value for lens decentration. A *P* value <0.05 was considered to be statistically significant.

## 3 Results

### 3.1 Baseline characteristics and results of orthokeratology for patients in both groups

A total of 54 eyes in 30 patients were analyzed for each group. Baseline characteristics were compared between the 2 groups including age and the degree and duration of myopia (Table 1). Two cases in the lens-decentration group and 3 cases in the control group failed to wear the orthokeratology lenses in the manner instructed.

There were 2 cases with ocular decentration after closing their eyes in the control group and 6 cases with physiological lagophthalmus in the lens-decentration group (Table 1).

It was noted that the direction of decentration was mainly temporal and inferior, which was consistent with previous reports (Table 1).^[11,13]^ There was no significant difference in best corrected visual acuity between the lens-decentration group and the control group (Table 1). However, considering the incidence of complications after orthokeratology, we recorded 2 cases of ghosting and 25 cases of corneal epithelial staining in the lens-decentration group, while no ghosting and only 7 cases of corneal epithelial staining were observed in the control group. These findings were consistent with previous reports (Table 1).^[11,14]^ It is noteworthy that no corneal central islands were detected in either group.

### 3.2 Comparison of the pretreatment corneal parameters between the 2 groups

A detailed comparison of the corneal parameters before orthokeratology is shown in Table 3. No significant differences were found in parameters such as horizontal and vertical corneal Q-value differences, vertical keratorefractive power (VK), E-values, corneal diameter, and average Q-values between the 2 groups (*P* > 0.05). However, in the lens-decentration group, it was found that horizontal keratorefractive power (HK) was much lower before treatment, while diopter and corneal Q-value differences between the nasal–temporal and superior–inferior quadrants (diameters ranging from 6 to 8 mm) were much higher compared to the control group (*P* < 0.05).

**Table 3:**
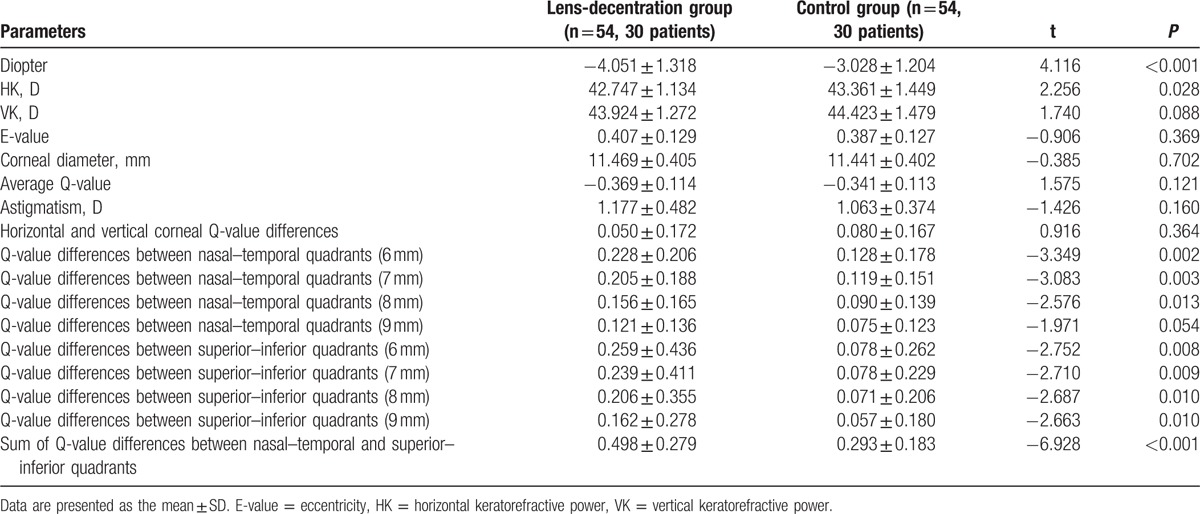
Comparison of the pretreatment corneal parameters between the 2 groups.

### 3.3 ROC curvature analysis of the pretreatment parameters to evaluate their predictive value for lens decentration

**Figure 1:**
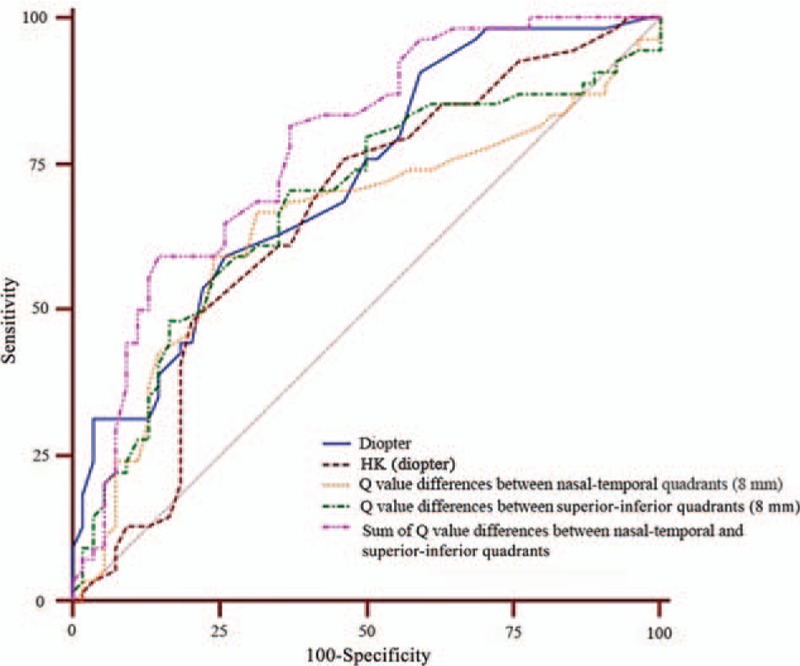
Area under the curve of the pretreatment corneal parameters.

ROC curvature analysis was conducted to evaluate quantitatively the predictive value of these pretreatment corneal parameters. As shown in Table 4 and Fig. 1, the area under the curve (AUC) of the sum of Q-value differences between nasal–temporal and superior–inferior quadrants (AUC = 0.778) was significantly larger than the AUC of the other parameters, including diopter, HK, horizontal and vertical corneal Q-value differences, Q-value differences between nasal–temporal quadrants (8 mm), and Q-value differences between superior–inferior quadrants (8 mm) (*P* < 0.001). Therefore, the sum of pretreatment corneal Q-value differences between nasal–temporal and superior–inferior quadrants was a reliable predictor for orthokeratology lens decentration; the satisfying cutoff value was 0.3 in the ROC analysis.

**Table 4:**
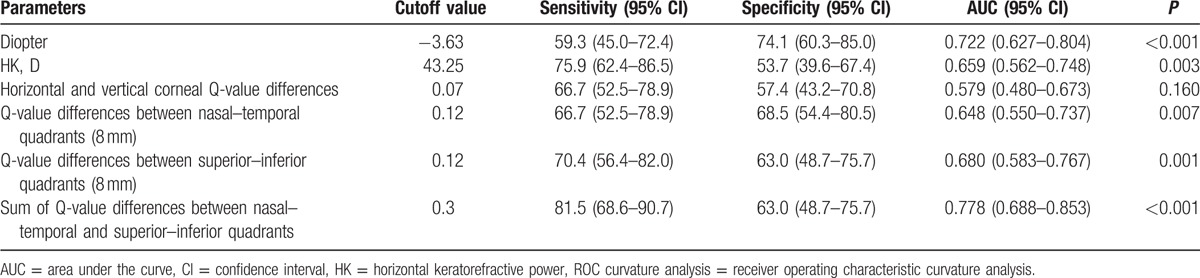
ROC curvature analysis of the predictive value of the pretreatment parameters.

## 4 Discussion

In the clinical application of orthokeratology, lens decentration causes complications which have become a problem for both physicians and patients.^[11,14]^ In the present study, it was confirmed that the temporal and inferior quadrants are the preferential sides for lens decentration in Chinese children and that this decentration is associated with the occurrence of complications such as ghosting and corneal epithelial staining. By analyzing various pretreatment corneal parameters, we further revealed much higher corneal Q-value differences between the nasal–temporal and superior–inferior quadrants in eyes with lens decentration than in those without lens decentration. By contrast, VK, HK, E-values, diameter, and astigmatism were comparable between the 2 groups. For the measurements of lens decentration, we used the optical zone around the corneal apex, which is in agreement with Atchison,^[15]^ who suggested that the corneal apex is a better reference position for the cornea center than the line of sight.

Our finding that eyes with lens decentration have higher Q-values in the temporal and inferior quadrants than their opposites provides a reasonable explanation for the cause of orthokeratology lens decentration. The Q-value was developed to describe the tendency for a change in corneal curvature from the central to the surrounding region and to measure quantitatively the Q-value of the corneal anterior surface.^[16]^ A single Q-value has been extensively used to represent the Q-value of the entire cornea. But Chen et al recently reported variations in the Q-values for different regions of the anterior corneal surface, indicating that a single value could not precisely reflect corneal Q-value.^[9,17]^ Consistent with this finding, we found that the average Q-value was comparable between eyes with and without decentration, but the corneal Q-value differences between nasal–temporal and superior–inferior quadrants were significantly higher in eyes with lens decentration, which indicated a more aspheric surface of the temporal and inferior quadrants. Importantly, this finding explains why orthokeratology lens prefer to shift to the temporal and inferior quadrants. In the clinic, it is often found that the lens easily shifts to the side with a high diopter value. Since patients wear orthokeratology lenses while sleeping, the lenses are relatively restricted and thus easily fixed to a more curved region of the cornea. Thus, with a corneal topography examination the following day, we could always detect lens decentration if it was present. Given that the alignment curve, which determines the stability of orthokeratology lens, is placed 8 mm from the corneal center, the Q-value difference at 8 mm between nasal–temporal and superior–inferior quadrants should be more significantly associated with lens decentration.

In addition, our data also provide some important insights into the individualized design of orthokeratology contact lens. Currently, the design of most orthokeratology lens only needs corneal diopter and curvature as well as a subjective refraction test. We found that the higher Q-value differences between the nasal–temporal and superior–inferior quadrants may directly cause lens decentration. However, despite being an important parameter for the degree of corneal Q-value, the corneal Q-value is not considered during the design of orthokeratology lens. Consequently, it is impossible to achieve a perfect match for the lens to an individual cornea. Therefore, giving a patient a corneal topography examination to determine the pretreatment Q-value differences between the nasal–temporal and superior–inferior quadrants, and adding these Q-values into the design of lenses, is critically important to improve the individualization of orthokeratology lens design, thus preventing lens decentration and improving the desired therapeutic efficacy.

Our preliminarily study has established a simple but effective method to predict lens decentration in patients receiving orthokeratology. ROC curve analysis showed that the sum of the Q-value differences between the nasal–temporal and superior–inferior quadrants is more sensitive than any other corneal parameters to predict lens decentration, with an AUC of 0.778 and a truncation point of 0.3. Through this predictive method, ophthalmologists can take effective measures to prevent lens decentration in vulnerable patients, which is particularly important when a completely individualized design of lens is not immediately available. One effective method to prevent lens decentration is to change the wearing protocol: patients first wear lenses in the daytime; as the eyes frequently blink, the lenses actively slide and make the corneal anterior surface symmetrical; lastly, patients wear the lenses during sleep.^[18]^

Thus, our study has shown that the high corneal Q-value difference between nasal–temporal and superior–inferior quadrants is an important cause of lens decentration toward the temporal and inferior quadrants of the eye. Based on such Q-value differences, a convenient but reliable method was developed to predict lens decentration. These findings demonstrate not only the importance of the corneal Q-value in the individualized design of orthokeratology lens but also allow ophthalmologists to take preventive measures for lens decentration in vulnerable patients. This will greatly reduce the occurrence of lens decentration and improve the efficacy and safety of orthokeratology. However, since a study by Fuller et al.^[19]^ revealed that Q-values showed different patterns between African-Americans and Whites, whether the findings of this study are applicable across diverse ethnicities will require further research.
